# Effect of Quadratus Lumborum Block in Patients With Acute-Subacute Unilateral Lumbar Strain

**DOI:** 10.7759/cureus.61014

**Published:** 2024-05-24

**Authors:** Emine Yıldırım Uslu

**Affiliations:** 1 Physical Medicine and Rehabilitation, Elazığ Fethi Sekin Şehir Hastanesi, Elazığ, TUR

**Keywords:** treating low back pain, low back pain, quadratus lumborum, low back pain (lbp), us-guided injection, quadratus lumborum block

## Abstract

Introduction: Lumbar strain originating from the quadratus lumborum (QL) is an important cause of low back pain; however, its diagnosis is often missed, and treatment is often inadequate. This leads to unnecessary diagnostic investigations and chronicization of pain. Therefore, it is important to treat it effectively and safely. In this study, we aimed to find out the effect of ultrasound (US)-guided QL block in acute-subacute low back pain caused by a strain of QL.

Materials and methods: Our study was retrospective, and the changes in the visual analog scale (VAS) and Oswestry Disability Index (ODI) scores within one week in 50 patients with acute-subacute localized low back pain, unilateral lumbar strain, palpation tenderness, paravertebral spasm, and decreased lumbar range of motion in the QL muscle, in whom we applied US-guided block to the QL muscle, were analyzed.

Results: There was a significant decrease in the mean VAS and ODI scores of the patients after the procedure. There was a negative correlation between improvement rates in VAS and ODI scores and age and body mass index (BMI). Recovery rates were higher in female patients than in male patients.

Conclusion: It can be said that US-guided QL block is an effective treatment method for QL-induced lumbar strains, and younger age, female gender, and lower BMI are associated with better responses after injection.

## Introduction

Low back pain is pain in the region between the 12th rib and the inferior gluteal fold and is a musculoskeletal system problem that is common in society, mostly caused by nonspecific causes and leads to disability [1]. The most common form of low back pain is nonspecific low back pain that cannot be attributed to a pathoanatomic cause [1]. Low back pain is categorized into three groups according to symptom duration: acute (< four weeks), subacute (4-12 weeks), and chronic (>12 weeks) [2]. Acute low back pain is quite common, and multifidus, iliocostalis, longissimus, and quadratus lumborum (QL) muscles may be the source of acute low back pain [3]. Low back pain of muscular origin is known as strain. QL, one of the spinal stabilizer muscles, is one of the most frequently missed muscle sources of low back pain [4].

The diagnosis of myofascial pain in the QL muscle is based on anamnesis and physical examination. The pain is usually felt deep and becomes more pronounced with movement. In some patients, pain radiation to the groin and trochanter major may be observed. Palpation of the muscle may result in tenderness and pain intensification [5]. Palpation of the muscle may reveal tenderness and pain. Spasms are quite common and increase the existing pain, and pain causes spasms. A vicious cycle of pain-spasm-pain occurs [6]. Breaking this cycle is important for an effective treatment. Injections may be effective in patients who do not respond to oral nonsteroidal anti-inflammatory drugs (NSAIDs) and myorelaxants, and in patients with lumbar motion limitation, corticosteroid injections are expected to selectively block the conduction of nociceptive fibers, and local anesthetics are expected to interrupt the pain and spasm cycle by relaxing intramuscular trigger points [7]. Fluoroscopy, computed tomography, and ultrasound (US) guidance have become widespread for safe and accurate injection. Recently, US has come to the forefront because it is a noninvasive, inexpensive, and effective technique [8].

Although lumbar strain originating from the QL is an important cause of low back pain, its diagnosis is often missed, and treatment is often inadequate. This leads to unnecessary diagnostic investigations and chronicization of pain. Therefore, it is important to treat it effectively and safely in the acute phase of pain. In this study, we aimed to find out the effect of US-guided QL block on pain and functional status in acute-subacute low back pain caused by a strain of QL.

## Materials and methods

This retrospective study was carried out in accordance with the principles of the Declaration of Helsinki, after receiving approval from the Fırat University Faculty of Medicine Ethics Committee (date: 11.03.2024; number: 22958). Informed voluntary consent forms were obtained from the participants. A total of 50 patients who underwent US-guided QL block at Elazığ Fethi Sekin City Hospital Polyclinics between 01.09.2023 and 29.02.2024 were included in the study.

Inclusion criteria

The inclusion criteria are as follows: 1) patients presenting with acute-subacute (less than 12 weeks) localized low back pain; 2) those who are between 18-60 years old; 3) tenderness, paravertebral spasm, and decreased lumbar range of motion are observed in the QL muscle with palpation; and 4) those who are unresponsive to oral medical treatments.

Exclusion criteria

The exclusion criteria are as follows: 1) those with accompanying lumbar discopathy and lumbar radiculopathy; 2) those with a history of malignancy, chronic disease, or inflammatory disease; 3) those who have coagulopathy or use anticoagulants; and 4) pregnant women.

The procedure was performed after sterile conditions were ensured. The paramedian sagittal oblique approach was preferred [9]. While the patient was in the prone position, the linear ultrasound probe was placed axially over the spinous processes of the lumbar vertebrae (at the level of L2) and then advanced approximately 3 cm laterally in the axial plane, the caudal part was turned slightly laterally, and the QL muscle was visualized. Using the in-plane technique, a 22 G needle was inserted at an angle of approximately 30 degrees from the lateral side of the probe. The fascia between the erector spinae and the QD muscle and the QD muscle were targeted. After negative aspiration, 2.63 mg betamethasone sodium phosphate + 5 mg betamethasone dipropionate + 4 cc prilocain mixture was injected into the QL muscle and fascia. After injection, patients were prescribed oral NSAIDs and thiocolchicoside.

The pain level of the patients was evaluated by the visual analog scale (VAS) score [10], and the effect of pain on daily life was evaluated by the Oswestry Disability Index (ODI) [11]. VAS and ODI results before and one week after the injection were analyzed. VAS and ODI scores before and after the procedure were compared. In addition, the percentage change of VAS and ODI scores before and after the procedure was calculated. The correlation of improvement percentages with age and BMI was analyzed.

Statistical method

Data were analyzed using the SPSS Statistical package (version 22.0; IBM, Armonk, NY). The characteristics of the variables were presented as a percentage, mean±standard deviation, and median. Wilcoxon, Student's t-test, and Pearson correlation tests were used. Statistical significance was accepted as p<0.05.

## Results

The study group consisted of 21 female and 29 male patients. The minimum patient age was 32 years, the maximum patient age was 60 years, and the mean age was 43.7±7.20 years. The minimum body mass index (BMI) was 20.36, the maximum BMI was 33.05, and the mean BMI was 26.35±3.20.

**Figure 1:**
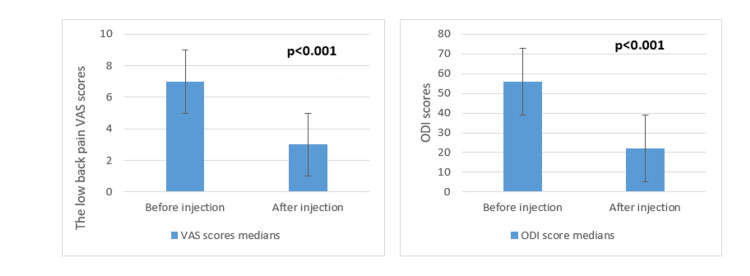
Change in VAS and ODI scores before and after the procedure VAS: Visual Analog Scale, ODI: Oswestry Disability Index

There was a significant decrease in the mean VAS and ODI scores after the procedure (Figure 1).

The correlation of the percentage change in VAS and ODI scores before and after the procedure with age and BMI was analyzed. A negative correlation was observed between age, BMI, and recovery rates (Table 1).

**Table 1:** Correlation analysis of the rate of improvement in VAS and ODI scores with BMI and age VAS: Visual Analog Scale, ODI: Oswestry Disability Index

	BMI		Age	
	r	p	r	p
VAS improvement rate	-0.566	0.000	-0.555	0.000
ODİ improvement rate	-0.466	0.001	-0.407	0.003

The rates of improvement in VAS and ODI values were higher in women than in men, and statistical significance was observed in the ODI score change (Table 2).

**Table 2:** Comparison of improvement rates in VAS and ODI scores in female and male patients VAS: Visual Analog Scale, ODI: Oswestry Disability Index

	Female, Male		
	Mean±SD	Mean±SD	p
VAS improvement rate	59.73±8.92	52.01±20.56	0.114
ODİ improvement rate	69.15±8.34	56.17±15.16	<0.001
n (%)	21 (42.0)	29 (58.0)	

All patients had a history of oral NSAIDs and myorelaxant use, and nine patients had a history of oral tramadol use before the procedure. It was determined that the patients did not need to use tramadol within one week after the procedure.

## Discussion

To the best of our knowledge, this is the first study to demonstrate the efficacy of QL block in acute-subacute low back pain caused by QL strains. Based on our study, it can be concluded that US-guided QL block is an effective treatment modality for QL-induced lumbar strains and that younger age, female gender, and lower BMI are associated with better responses after injection.

Myofascial pain of the QL muscle is among the most common musculoskeletal pathologies in patients with low back pain [12]. Although the exact function of the QL is unknown, it has been reported that it acts as an agonist muscle during trunk extension and lateral flexion and stabilizes the spine during flexion [13]. The QL muscle consists of iliocostal, iliolumbar, iliothoracic, and lumbocostal components [14]. Myofascial pain in the deep fibers of the muscle extends to the sacroiliac joint and the lower part of the hip, while pain in the superficial fibers may extend from the crista iliaca to the trochanter major [15]. It usually does not cause pain radiating to the leg and paresthesia. However, if combined sacroiliac joint lesions or gluteus minimus trigger points are present, it may mimic disc radiculopathy and may be responsible for failed back surgery [12]. Therefore, lumbar muscles, especially the QL muscle, should always be included in the differential diagnosis for accurate diagnosis and effective treatment of low back pain.

The efficacy of manual therapy techniques such as transcutaneous electrical nerve stimulation and muscle energy technique and strain counterstrain technique in myofascial pain of the QL muscle, has been examined in various studies, and it has been suggested that they are effective [16,17]. Interventional procedures are known to be more successful than conservative options in the treatment of myofascial pain syndrome; these procedures include the administration of injection agents, such as botulinum neurotoxin, lidocaine, steroids, and saline. However, an optimal injection point for the QL muscle has not been determined [18].

Ultrasound-guided QL block is a recently described type of block in which local anesthetic is injected into the QL muscle and the fascia surrounding the muscle to numb the thoracolumbar nerves [8]. It is widely used for postoperative analgesia [19]. However, there are limited studies investigating its efficacy in the treatment of low back pain. Barreto et al. [20] showed that levobupivacaine and triamcinolone injection into the QL muscle was effective for six months in chronic myofascial low back pain. Andres et al. [21] concluded that botulinum toxin was not superior to bupivacaine in the treatment of myofascial pain in the iliopsoas and QL muscles. Hong et al. [22] compared the efficacy of US-guided trigger point injection and extracorporeal shock wave therapy (ESWT) in QL myofascial pain and found ESWT superior. To the best of our knowledge, there is no study investigating the effect of QL block in acute low back pain caused by QL strains. In our study, QL block was found to be effective in acute-subacute low back pain in terms of pain and functionality in the early period. In addition, younger age, female gender, and lower BMI were associated with better responses after injection. It was thought that the negative correlation between age and recovery rates may be related to the increased frequency of concomitant lumbar degeneration at advanced age, and the negative correlation between BMI and recovery rates may be related to the fact that it is easier to reach the deep points of the muscle in patients with low BMI [23].

The limitations of our study include the lack of a control group, the fact that the pain threshold levels of the participants were not evaluated, the long-term results of the injections could not be evaluated and were limited to one week, and the patients used oral NSAIDs and myorelaxants after injection.

## Conclusions

It can be said that US-guided QL block is an effective treatment modality for QL-induced lumbar strains whose diagnosis is often missed and may result in chronic pain due to inadequate treatment; younger age, female gender, and lower BMI are associated with better responses after injection. However, prospective, randomized, long-term clinical trials, including placebo groups, are needed to determine its efficacy and safety with more definitive results.
